# A review of wearable motion tracking systems used in rehabilitation following hip and knee replacement

**DOI:** 10.1177/2055668318771816

**Published:** 2018-06-18

**Authors:** Shayan Bahadori, Tikki Immins, Thomas W Wainwright

**Affiliations:** Orthopaedic Research Institute, 6657 Bournemouth University, Bournemouth, UK

**Keywords:** Total knee replacement, total hip replacement, rehabilitation, wearables

## Abstract

Clinical teams are under increasing pressure to facilitate early hospital discharge for total hip replacement and total knee replacement patients following surgery. A wide variety of wearable devices are being marketed to assist with rehabilitation following surgery. A review of wearable devices was undertaken to assess the evidence supporting their efficacy in assisting rehabilitation following total hip replacement and total knee replacement. A search was conducted using the electronic databases including Medline, CINAHL, Cochrane, PsycARTICLES, and PubMed of studies from January 2000 to October 2017. Five studies met the eligibility criteria, and all used an accelerometer and a gyroscope for their technology. A review of the studies found very little evidence to support the efficacy of the technology, although they show that the use of the technology is feasible. Future work should establish which wearable technology is most valuable to patients, which ones improve patient outcomes, and the most economical model for deploying the technology.

## Background

Total knee replacement (TKR) and total hip replacement (THR) are highly successful operations for controlling pain, restoring function, and enhancing quality of life for patients with hip and knee osteoarthritis.^1–3^ They are amongst the most common surgical procedures worldwide.^4^ However, approaches to rehabilitation following surgery vary greatly and evidence is limited with regard to successful interventions.^5^ The introduction of enhanced recovery after surgery protocols to improve post-surgical recovery has reduced hospital length of stay^6,7^ for THR and TKR patients, with recent studies indicating that same day discharge is feasible.^8^ This decrease in time for inpatient rehabilitation post-surgery highlights the need for guidance for patients on rehabilitation once home, particularly as recent research has shown that physical activity does not increase following THR or TKR.^9^ Innovative methodologies such as the use of Actigraph data^10^ are now available to assess specific activity intensity post-surgery and so enable the evaluation of the use of wearable technologies as part of a suitable programme that empowers patients to complete physiotherapy at home.^2^ Traditionally patient adherence to recommended home-based physiotherapy programmes is poor. For example, only 24% of patients with osteoarthritis were found to comply with their exercise programme.^11^ A lack of time,^12^ failure to remember how to do the exercises,^13^ limited understanding of how the programme makes them better,^14^ and a lack of feedback^15^ are some of the barriers to patients being more compliant.

The economic burden from both direct and indirect associated costs of rehabilitation post THR and TKR in the National Health Service is also a major consideration. Commonly physiotherapy is not provided post discharge for THR and only occasionally provided for TKR, mostly within a group setting.^16^ Therefore, proving cost neutrality or effectiveness against current practice may be difficult when introducing additional technology or cost to rehabilitation. This is the economic reality and so research needs to demonstrate improvement to clinical outcomes and provide a proven business case for adoption.

Recently, there has been a proliferation of devices designed to monitor activity, educate patients, and provide feedback following TKR and THR surgery.^2^ Their aim is to develop the relationship between physiotherapist and patients, and increase exercise adherence.

In broad terms, patient monitoring can be categorised into five types of system:

- Classic mechanical systems, e.g. contact angle goniometer;
- Markerless motion capture tracking systems, e.g. Microsoft Kinect™;
- Marker-based optical motion technologies, e.g. Vicon™ ;^17^
- Robot-assisted rehabilitation
- Wearable tracking systems.^18^

The goal for all of these systems is to deliver better care at lower cost to patients and improve patient outcomes.^19^ This review focuses on wearables tracking systems.

There are three types of platforms used by wearable devices, and indeed many devices use all three platforms:^20^

- Physiological sensing: These systems have sensors capable of detecting and quantifying force, motion, displacement, and vibration from internal biological functions;^21^
- Communication interface: This is in the shape of hardware or software to collect physiological and motion data;
- Data interpretation techniques: These extract clinically relevant information from physiological and motion data.

There is a wide variety of wearable devices currently being marketed, which are proposed to assist with rehabilitation following joint replacement. However, very little is known about how these technologies work, how they differ, and whether they are effective. The aim of this review is to provide an overview of wearable devices available for hip and knee replacement rehabilitation and assess the evidence on whether they do improve outcomes for patients.

## Method

### Literature search strategy

This review is reported according to the Preferred Reporting Items for Systematic Reviews and Meta-Analyses statement (www.prismastatement.org/PRISMAStatement). A computer-based search was completed in October 2017 using the mySearch Database (Bournemouth University). This included Cochrane Database of Systematic Reviews library, CINAHL Complete®, Science Citation Index, and Medline®.

Articles published in the English language from January 2000 to October 2017 were reviewed. Search strategy terms are outlined in Table 1.

**Table 1.** Literature search strategy.

Patient	(MM “Arthroplasty, Replacement, Hip”)
	(MM “Hip Prosthesis”)
	(Hip*) N5 (arthroplast* OR prosthes* OR replace*)
	(MM “Arthroplasty, Replacement, Knee”)
	(MM “Knee Prosthesis”)
	(Knee*) N5 (arthroplast* OR prosthes* OR replace*)
	AND
Rehabilitation	Rehabilitat* OR Recovery
	AND
Wearable Systems	Tracker*
	Device*
	Wearable*
	Sensor*

MM (MeSh term). “” used to find exact phrase. *used to find all word with a common stem. N5 to find all articles containing the keywords within five words.

**Figure 1.**
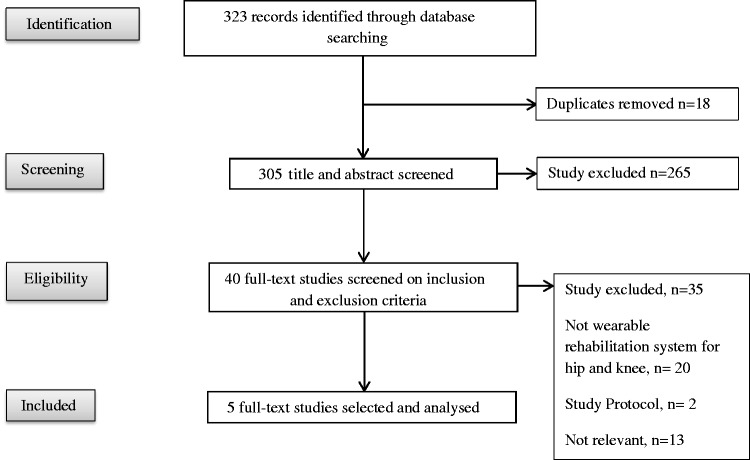
Prisma flow chart of results from the literature search^48^.

Once the initial searches were completed, the results were manually filtered to remove duplicates. Three independent reviewers (SB, TWW, and TI) then screened journal titles and abstracts for relevance until only 40 papers remained (see Figure 1 for flow chart). SB and TW then assessed the full text of the papers, and five papers were found to meet the eligibility criteria. Any disagreements between reviewers were discussed with TI and resolved by consensus. Studies included were portable wearable technologies capable of providing feedback to the end user following hip or knee replacement surgery.

### Data extraction process

SB extracted data to a prearranged standardised table. The table template included the study reference, study population, technology used, how it worked, wearability and placement of technology, aim of the study, analysis, and outcomes (Table 2).

**Table 2.** Article summary.

Reference	Study population	*Device* Technology	What does it do?	Wearability and placement	Aim of study	Analysis	Outcomes of study
Chiang et al.^23^	TKR patients (n = 18).	***APDM***Sensor (accelerometer, gyroscope)	Monitors knee ROM	Strapped with Velcro on anterior side of thigh and shin	To propose a method to conduct data collection and analysis for recovery progress of ROM using wearable sensors	Correlation analysis between knee ROM and BMI, use of pain control, and haemostatic agent used. Questionnaire on patient perception of device.	Results suggest that BMI, use of pain control, and haemostatic agents are related to recovery progress of knee ROM in TKR. Wearable device can provide alternative to traditional goniometer measurements.
Gonzalez-Franco et al.^24^	Healthy participants (n = 16).	***Wocket, UNITY*** Virtual Reality open source gaming sensor	Physiotherapy monitored and taught through gaming interface using avatar trainers	Strapped with Velcro, around the thigh and the lower leg	To evaluate a prototype rehabilitation system for TKR	Average performance scores calculated of six exercises repeated in two sets, and participants completed questionnaire on learning, usability, attention demand, interactions, agency, time, and pain perception.	A significant improvement from the first set to the second one for both familiar and unfamiliar exercises, suggesting system enabled participants to learn. All participants were able to start, follow, and finish whole therapy without external intervention.
Jeldi et al.^25^	THR patients (n = 44).	***ActivPAL3***Sensor (accelerometer)	Monitors physical activity (PA)	Attached with adhesive patch on anterior aspect of thigh	To gain insight into mobilisation, upright times, and sit-to-stand transitions in hospital	Outcomes from the sensor data were calculated for the total post-surgery hospital stay, first 24 h post-surgery, last 24 h before discharge, and time associated with physiotherapy or occupational therapy. Compared by gender (13 males, 31 females).	During first 24 h considerable variation in STS, total upright time, and longest upright bout. This variation continued to last 24 h before discharge, where males had longer upright bouts than females.
Kwasnicki et al.^26^	TKR patients and healthy volunteers (n = 29).	***e-AR***Sensor (tri-axial Micro-Electro-Mechanical-System (MEMS) accelerometer)	Assesses mobility	Worn on ear	To assess feasibility of using a wearable sensor to monitor patient recovery in a community setting	Perioperative data for TKR patients compared with healthy volunteers **based on activity protocol derived from Dynaport® Knee Test.** Sensor data compared for TUG and ROM scores; gait assessed through analysis of sensor data; and similarities in performance at specific times reviewed.	Patient function estimated by sensor consistently declined following TKR before returning to near normal function after 24 weeks. Healthy volunteers and patients at same peri-op stage seen to exhibit similar kinematic profiles.
Lin and Kulić^27^	THR and TKR patients (n = 7)	***SHIMMER*** Sensor (accelerometer, gyroscope)	Estimates joint ROM	Strapped with Velcro on knee, hip, and ankle	To evaluate performance of a joint estimation algorithm that utilises strap-on IMU sensors in clinical rehabilitation setting.	Patients’ exercises were monitored from day one to discharge	Initial results demonstrated promise for use in clinical setting. Accelerometer RMS error of 0.40 m/s^2^ and a gyroscope RMS error 0.09 rad/s.

### Data quality

The Risk Of Bias In Non-randomized Studies – of Interventions (ROBINS-I)^22^ tool was used to assess the risk of bias. The assessment includes seven domains including confounding, selection of participants into the study, classification of interventions, deviations from the intended interventions, missing data, measurement of outcomes, and selection of the reported result. The categories for risk of bias judgements for ROBINS-I are ‘low risk’, ‘moderate risk’, ‘serious risk’, and ‘critical risk’ of bias.^22^

## Results

### Classification of technologies and physiotherapy applications

Chiang et al.^23^ introduced a tracking device **(APDM****^28^****)** for measuring range of motion (ROM) following TKR using a sensor, which is a usually a combination of accelerometer, gyroscope, barometer, magnetometer, and a temperature sensor. For this research, only the accelerometer and gyroscope were active and were placed on the thigh and shin. Knee ROM was calculated using sensor data following stretching and walking exercises. This feasibility study examined the correlation between knee ROM and patient body mass index, use (or not) of epidural patient control anaesthesia, and type of haemostatic agent used, at various time points before and up to six weeks following surgery. They found an association between these three factors with the recovery progress of knee ROM following TKR. They also found that 83% of patients did not find the sensor belt used uncomfortable.

A study by Jeldi et al.^25^ measured upright time (UT) and sit-to-stand (STS) transition progression after THR. Using an accelerometer sensor **(ActivPAL3^29^)** attached to anterior aspects of the non-operated thigh, patients were monitored for their post-surgery in-hospital stay. Data output from the sensor showed considerable variation in the STS results for the first 24 h. Similarly the last 24 h did not follow any pattern for STS or UT. Results showed the female patient stay to be on average 20 h longer than for male patients, and female patients also performed less STS and UT in the first and last 24 h. In some cases, data collection was affected by the post-surgery side effects such as low blood pressure, nausea, vomiting, and individual health-related problem. Nevertheless, the wearable sensor was able to collect all data related to STS and UT for the duration of the hospital stay, providing insight into patient recovery and response to rehabilitation post THR.

Kwasnicki et al.^26^ aimed to investigate the feasibility of using an ear-worn motion sensor **(e-AR****^30^****)** to conduct objective, home-based mobility assessments in the perioperative setting. The sensor contained a triaxial micro electro mechanical system accelerometer with data monitored remotely using a tablet computer by a health care professional. Patient mobility was derived from sensor motion data creating a kinematic impression of participant movement. The activity protocol was divided into four sections and baseline data from a healthy participant were used for comparison. The four sections were walking; stepping up and down; picking up an object and walking, or sitting down and standing up; and lifting and moving an object. Results found that measuring patient mobility was feasible in the community setting. Overall, the motion sensor measurements were consistent in repetitive tasks, left/right symmetry, and magnitude of linear acceleration and it was feasible for the wearable to record information close to daily activities compared to one-dimensional TUG or STS movements. However, the average age of healthy participants was a lot younger compared to study cohorts and therefore caution should be taken with reference to a direct comparison.

A study by Lin and Kulić^27^ used IMU sensors **(SHIMMER****^31^****)** to collect patients’ movement data and combined it with a kinematic model to estimate the joint angles. The motions performed by various TKR patients were the exercises prescribed by their physiotherapist based on the assessment of the patients’ progress. Designed as a proof of concept study, only seven patients were recruited and data collected from the sensor were validated against data from healthy participants. Joint angle and angular velocity performing extension–flexion, abduction–adduction, and internal rotation were recorded using the sensor. Root mean square error for sensor data and also key pose (initial stationary pose) error was calculated for both health and joint replacement patients. Reported outcomes showed similar errors on average, and the authors concluded that their system was valid in a clinical setting for joint replacement patients undergoing physiotherapy.

Gonzalez-Franco et al.^24^ used a sensor-enabled virtual reality gaming open-source platform using **Wocket****^32^**** and UNITY****^33^** to address the problem of patient adherence to physiotherapy following TKR. In the game, participants followed on-screen instructions to perform physiotherapy. The protocol comprised two sets of three familiar and three unfamiliar exercises, each with 10 repetitions. An avatar (virtual trainer) demonstrated each exercise, and a second avatar examined the quality of the performed exercise based on velocity and knee angles achieved. The authors found that the interface motivated participants to complete their exercises, and that participants were able to learn and improve just by doing the exercises, without further human intervention. Participants were positive about the system in relation to their interactions, learning, control, pain perception, attention demanding, usability, and time perception, with responses to a questionnaire being measured on a 10-point Likert scale.

No adverse effects were reported for any of the devices utilised in the studies reviewed; however, the quality of reporting in the papers was variable and this should be taken into account when reviewing the evidence. All five studies reviewed used an accelerometer and a gyroscope for their technology, with the aim to assess the feasibility of using a wearables sensor to monitor, evaluate, and educate patients’ recovery. Initial results demonstrate promise for use of these devices in clinical settings.

### Risk of bias

Assessed using the ROBINS-I risk of bias tool, all reviewed studies were judged to be at serious risk as there were bias issues in more than one domain.

## Discussion

### Clinical assessments and the evidence of use

The main goal of wearable devices for rehabilitation is to capture movement and posture of patients for monitoring their motor activities during rehabilitation therapy. Clinical trials are crucial to assess the success of the new technologies, in particular when additional clinical results show improvement in patient condition. Post-operative monitoring with wearable technologies has already been examined clinically in patients undergoing spinal surgery,^34^ stroke, and arm rehabilitation.^35–37^ Reported outcomes show excellent overall patient satisfaction. Hadjidj et al.^17^ also outlined the innovation technologies currently used in enhanced recovery surgical programmes such as wireless and contact free sensors for monitoring functional recovery and improving post-surgical recovery using wearable sensors.

The search did not find any papers adopting randomised trials to assess the technology for rehabilitation post hip and knee replacement. As discussed the studies included in our review were small, feasibility studies, of varying quality, therefore they were not generalisable. Reviews on upper body wearable rehabilitation systems^7,38^ have found very little evidence as yet to support the use of the devices. This may be because of the length of time that is required for developing a new technology, or because predeveloped or early stage systems do not justify the time consuming and costly process of clinical trials. It is also important to acknowledge that the biggest challenge for TKR and THR wearable rehabilitation devices may be that the optimal rehabilitation pathway is yet to be defined,^39^ therefore the question of what programmes rehabilitation wearables should help to facilitate and deliver remains unanswered.

It is worth noting that none of the studies examined or reported on the health economics of introducing the technology or on the longer term benefits to outcomes such as Patient Reported Outcome Measures using this technology. Even if evidence is collected that supports the clinical benefit of wearable devices, if there is not a sustainable business case for their use, they are unlikely to be widely adopted in health care systems.

Interestingly, devices used in the studies reviewed here have also been marketed to have the potential to measure heart rate variability in anorexic patients,^40^ analyse cardiac health^41^ by capturing the contextual and metabolic information of the user, monitor stroke patients’ physical activity,^42^ and assess functional mobility in patients with neurological disorders. A study on the latter^43^ uses the same sensor as that employed by Chiang et al.,^23^ and initial findings in a small randomised trial were positive.

In contrast to the focus of this paper which was to examine whether the devices reviewed can improve patient outcome reports post THR and TKR, it could be argued that the features of the motion tracking monitors such as the sensor location and placement are factors that should be included in the evaluation of the effectiveness of wearable devices. Evaluating whether these devices improve outcomes for patients is complex as the wearable monitoring platforms provide feedback information as well as coaching to the patients. The Gonzalez Franco et al.^24^ paper is the only paper here that evaluates both the feedback and the coaching provided. It should be noted that the participants in the Gonzalez Franco paper were healthy, and not patients following hip or knee replacement, as stated in the search criteria. However, the wearable was designed to be used by patients following knee replacement so the authors felt that its inclusion was of value to the study.

New possibilities are rising with the use of smartphones and applications to estimate joint angles,^44^ as well as the potential of exciting upcoming technologies such as nano-sensors and e-textiles.^45^ It is important that further research is done to study their efficacy, and indeed study protocols are now being published for larger randomised controlled trials using wearable technologies for post TKR patients.^46,47^ The studies included in this review demonstrate that the technology is safe and feasible and that it shows promise. It is also popular with patients which is likely to drive research and development in this area.^24^

## Conclusion

Wearable technology is being promoted by companies as a way of improving rehabilitation following THR and TKR surgery. However, this review finds very little evidence to support its efficacy. The small numbers of studies do, however, show it is feasible, and like most new technology, including patient/technology interfaces, it will improve over time. Future work should establish which wearable technology is most valuable to patients, which ones improve clinical outcomes, and what are the best economical models for their deployment.
